# Array of Metabolites in Italian *Hericium erinaceus* Mycelium, Primordium, and Sporophore

**DOI:** 10.3390/molecules24193511

**Published:** 2019-09-27

**Authors:** Federica Corana, Valentina Cesaroni, Barbara Mannucci, Rebecca Michela Baiguera, Anna Maria Picco, Elena Savino, Daniela Ratto, Claudia Perini, Hirokazu Kawagishi, Carolina Elena Girometta, Paola Rossi

**Affiliations:** 1 Centro Grandi Strumenti, University of Pavia, 27100 Pavia, Italy; federica.corana@unipv.it (F.C.); barbara.mannucci@unipv.it (B.M.); 2 Department of Earth and Environmental Sciences, University of Pavia, 27100 Pavia, Italy; valentina.cesaroni01@universitadipavia.it (V.C.); rebeccamichela.baiguera01@universitadipavia.it (R.M.B.); annamaria.picco@unipv.it (A.M.P.); elena.savino@unipv.it (E.S.); 3 Department of Biology and Biotechnology “L. Spallanzani”, University of Pavia, 27100 Pavia, Italy; daniela.ratto@gmail.com (D.R.); paola.rossi@unipv.it (P.R.); 4 Department of Life Sciences, University of Siena, 53100 Siena, Italy; claudia.perini@unisi.it; 5 Research Institute of Green Science and Technology, Shizuoka University, Shizuoka 422-8529, Japan; kawagishi.hirokazu@shizuoka.ac.jp

**Keywords:** medicinal mushroom, *Hericium erinaceus*, bioactive compounds, mycelium, sporophore, primordium, erinacines, hericenones, hericenes

## Abstract

*Hericium erinaceus* is a medicinal mushroom that contains many molecules promising a plethora of therapeutic properties. In this study, the strain H.e.2 (MicUNIPV, University of Pavia, Italy) was isolated from a sporophore collected in Tuscany (Italy). Mycelium, primordium, and wild type and cultivated sporophores were analyzed by HPLC-UV-ESI/MS. Erinacine A in the mycelium and hericenones C and D in the sporophores were quantified by comparison with their standard molecules. For the first time, *H. erinaceus* primordium was also investigated for the presence of these molecules. Comparing with the literature data, hericenes, molecules structurally similar to hericenones, were present in all our samples. The highest contents of hericenones C and D were detected in cultivated sporophores, compared to the wild type. The comparison of these data with those of another Italian *H. erinaceus* strain (H.e.1 MicUNIPV) was discussed. The results led us to select *H. erinaceus* strains more suitable for mycelium production or sporophore cultivation to obtain extracts with a higher content of bioactive compounds. This work provides a further step towards standardizing the procedures in the development of dietary supplements made from mushrooms.

## 1. Introduction

*Hericium erinaceus* (Bull.) Pers. is a fungus belonging to Basidiomycota, Agaricomycetes, Russulales, and Hericiaceae [1]. Among all mushrooms, *H. erinaceus*, an edible and medicinal mushroom in traditional Chinese medicine, has been widely reported to have healthy effects on: the central nervous system [2,3]; different cancerous cell lines, such as HepG2 (hepatoma), MCF7 (breast cancer), HL-60 (human acute promyelocytic leukemia), and SGC-7901 (human gastric cancer cells) [4,5,6]; depression [7]; diabetes [8]; lipedema [9]. It also exhibited a reversion of frailty cognitive decline during aging [10]. Up to now, about 70 different secondary metabolites have been isolated from either sporophore or mycelium, or both. The investigation of chemical constituents promising for their properties is constantly being updated in the search for the discovery of a new drug source [11]. Both the high-weight metabolites (e.g., polysaccharides) and low-weight metabolites (e.g., polyketides, phenols, and terpenoids) include bioactive molecules, although each substance category provides in turn an extremely various bouquet of molecules, where only a fraction shows evidence for bioactivity [12,13,14,15]. Hericenones are low-weight aromatic compounds first isolated by Kawagishi et al. (1990) [14] from the sporophore of *H. erinaceus.* Up to now, eight different compounds have been recognized as hericenones (A–H) [11,16]. Hericenones C, D, and E have stimulating activity on the synthesis of nerve growth factor (NGF) [17]. Hericenone F has been reported to be responsible for an anti-inflammatory effect by reducing nitrogen monoxide (NO) release [18]. Analyses of dried sporophores have detected different volatile compounds, some of them represented by hexadecanoic acid, linoleic acid, phenylacetaldehyde, and benzaldehyde. The erinacines in *H. erinaceus* are a group of cyathane-type diterpenoids, including 20 members of 24 diterpenoids described by Tang et al. [19]. To date, 15 erinacines (A, B, C, D, E, F, G, H, I, P, Q, J, K, R, S) isolated from *H. erinaceus* mycelium have been identified and eight out of 15 show neuroprotective properties, such as enhancing NGF release (erinacines A–I), reducing amyloid-β deposition, increasing insulin-degrading enzyme (IDE) expression (erinacines A and S), and managing neuropathic pain (erinacine E), while the others have different pharmacological activities [12,20,21,22,23,24,25,26].

At present, *H. erinaceus* is widely used as a dietary supplement. Nevertheless, the lack of standardization strongly affects the quality and effective bioactivity of the final product. As above described, only a few molecules have been reported to stimulate NGF release, namely erinacines A–I from mycelium (the most studied being erinacine A) and hericenones C–D from sporophore. To obtain this specific target on neuroprotection, the standardization process of dietary supplements therefore relies on the selective detection and quantification of such molecules. A major problem at this concern is the availability of pure analytical standards, due to the difficulty in the isolation and achievement of suitable amounts. 

The aim of this study is to analyze and compare different stages of *H. erinaceus* (mycelium, primordium, and sporophore) sampled in Tuscany (Italy), in order to detect the presence and to quantify the concentration of the target bioactive metabolites erinacine A and hericenones C and D. The results could be useful for suggesting optimization strategies for future dietary supplements.

## 2. Results

### 2.1. H. erinaceus Samples for Chemical Analyses

The *H. erinaceus* wild type (WT) sporophore analyzed in the present study was collected on a living holm oak (*Quercus ilex* L.) in the hilly area around Siena (Italy) in 2018. The sporophore was identified based on the macro- and micromorphological characteristics of the species [27]. The main features are reported in Table 1.

The strain obtained from the WT was confirmed to belong to *H. erinaceus* [28] and is maintained in the Fungal Research Culture Collection of Pavia University (MicUNIPV) as H.e.2.

The cultivation of *H. erinaceus* at the Botanical Garden of the University of Pavia (Italy) led to the collection of 44 sporophores, total weight 1344.2 g. The diameter of the collected samples was 6–15 cm.

**Figure 1:**
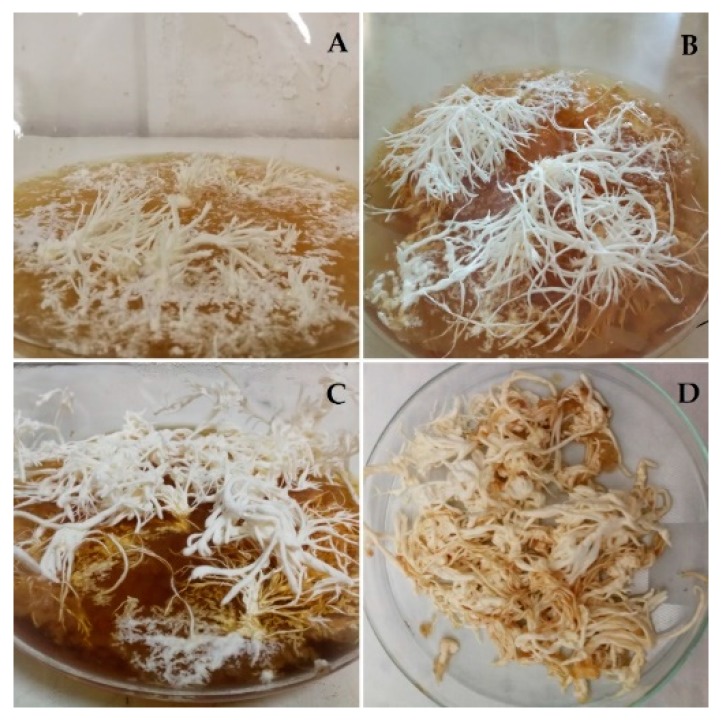
Different stages of growth of the primordium. 30 (**A**), 45 (**B**), 60 (**C**) days of growth and fresh collected material (**D**).

The primordium can be considered the transition stage before sporophore. It is formed by dense mycelial cords developing with negative geotropism (Figure 1). Primordia were harvested after 60 days, at the maximum of their development, and the fresh material was analyzed.

### 2.2. Chemical Analyses

We processed 1 g of lyophilized mycelium, dried WT and cultivated sporophores, and fresh primordium for chemical analyses.

In order to identify and analyze the bioactive metabolites present in our samples, we compared them with the standard molecules of erinacine A and hericenones C and D by HPLC-UV-ESI/MS. The molecular formula, chemical structures, and molecular weight of these molecules are reported in Table 2 [29].

**Figure 2:**
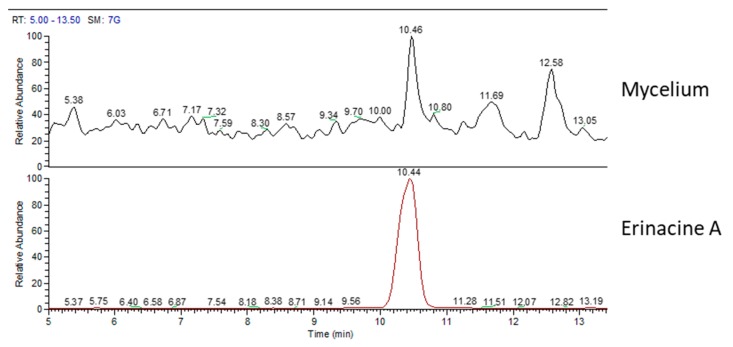
Mass spectrum (MS) traces of mycelium (top) and erinacine A molecule standard (bottom).

Figure 2 shows the mass spectrum (MS) chromatographic traces of H.e.2 mycelium and the standard molecule of erinacine A. The standard erinacine A was detected using HPLC-UV-ESI/MS at the retention time (RT) of 10.44 min. By comparing the RT and molecular ion or mass spectra, the presence of this molecule in the H.e.2 mycelium was detected too. Besides, the chromatographic trace of H.e.2 mycelium also showed a peak at RT 12.58 min that belongs to a molecule not yet identified with a MW of 430 Da.

**Figure 3:**
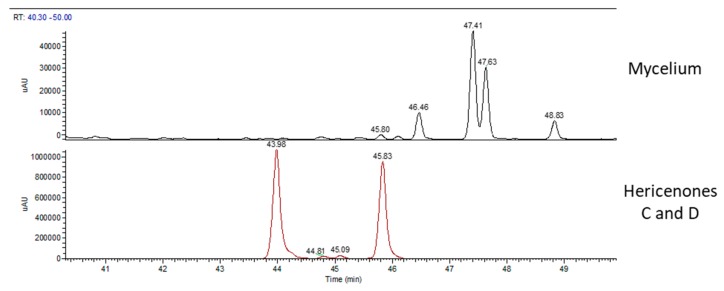
UV traces of mycelium (top) and the standard molecules of hericenones C and D (bottom).

Figure 3 shows the UV chromatographic traces of H.e.2 mycelium and the standard molecules of hericenones C and D. In mycelium, hericenones C and D were not present. Moreover, the peaks at RT 46.46 min, 47.41 min, 47.63 min, and 48.83 min were supposed to be, respectively, hericene D, hericene A, hericene B, and hericene C, based on the data reported by Arnone et al. (1994) and Kobayashi et al. (2018) [30,31]. The molecular formula, chemical structures, and molecular weights of the hericenes are reported in Table 3 [29]. 

**Figure 4:**
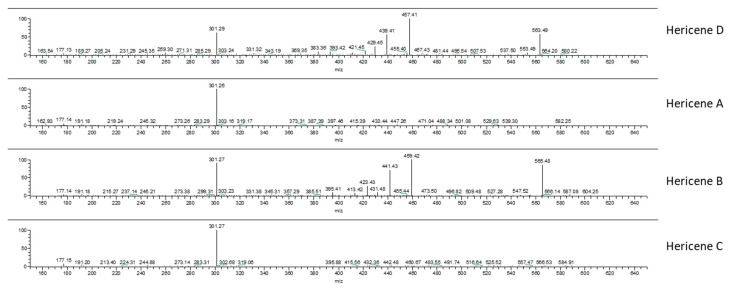
MS/MS-ESI spectra of hericenes D, A, B, and C (from top to bottom).

Our hypothesis was also confirmed by ion fragments in MS/MS spectra. Figure 4 reports the MS/MS-ESI spectra of hericenes: from top to bottom, hericenes D, A, B, and C are shown. The ion *m*/*z* 301 was present in all the spectra and derives from the loss of the side chain R. Spectra of hericenes D and B also show ions derived from fragmentation close to double bonds of R.

WT and cultivated sporophores were analyzed. The data were compared with the standard molecules hericenones C and D. 

**Figure 5:**
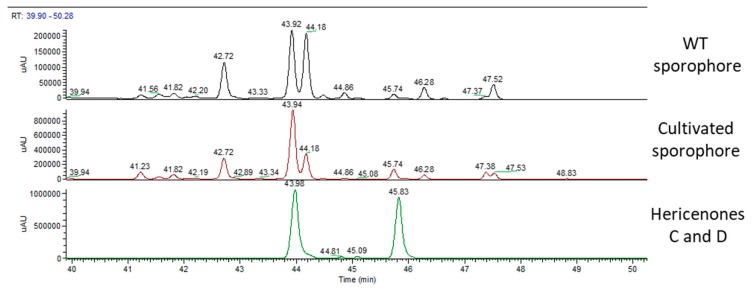
UV traces of wild type sporophore (top), cultivated sporophore (middle), and hericenones C (RT 43.98 min) and D (RT 45.83 min) standards (bottom).

Figure 5 shows the UV chromatographic traces of the WT sporophore collected in Italy, the cultivated and the two standard molecules of hericenones: C detected at RT of 43.98 min and D detected at RT of 45.83 min. Hericenone C was detected in the WT at RT of 43.92 min and at RT of 43.94 in the cultivated sporophore. Hericenone D was detected at RT of 45.74 min in both samples. There were also other peaks close to these hericenones that could be attributed to hericenone E (RT of 42.72 min), hericenone I (RT of 44.18 min), and hericenone H (RT of 47.37 min) on the basis both of previous publications [16] and the similarity of their fragmentation pattern to that of hericenones C and D standard. In the same chromatogram (Figure 5), there were peaks that could be attributed to hericenes: from lower to higher RT hericene D (RT 46.28 min), hericene A (RT 47.37 or 47.38 min), hericene B (RT 47.52 or 47.53 min), and hericene C (RT 48.83 min). 

**Figure 6:**
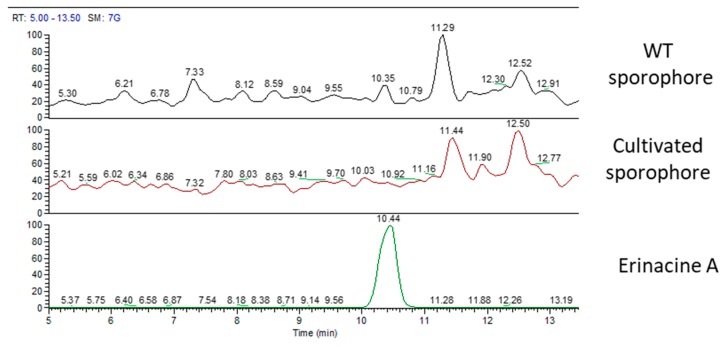
MS traces of wild type sporophore (top), cultivated sporophore (middle), and erinacine A standard molecules (bottom).

Figure 6 displays MS chromatographic traces of the two sporophores and the standard molecule of erinacine A. The erinacine A molecule was not present in both sporophores. Besides, the chromatographic traces also showed other peaks at RT of 11.29 and 12.52 min for the WT sporophores and at 11.44 and 12.50 min for the cultivated sporophores that belong to molecules not yet identified.

**Figure 7:**
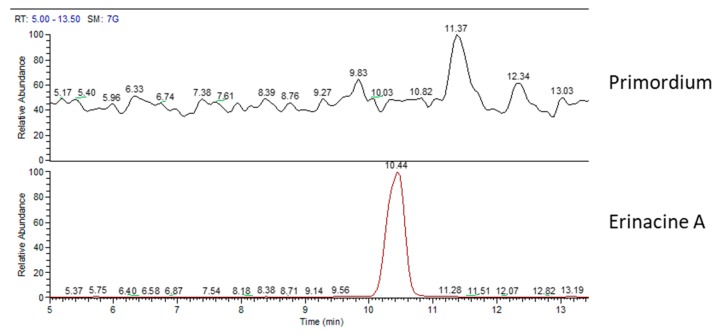
MS (mass spectrum) traces of primordium (top) and erinacine A molecule standard (bottom).

**Figure 8:**
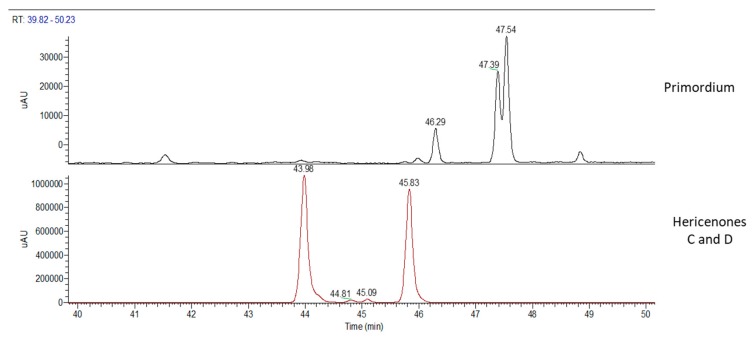
UV traces of primordium (top) and hericenones C and D standards (bottom).

Chemical analyses of primordium showed neither erinacines (Figure 7) nor hericenones (Figure 8). MS trace of primordium (Figure 7) also showed a peak at RT of 11.37 min that has not yet been determined. Instead, the UV trace of primordium showed peaks at the same RT described for hericenes D, A, B, and C (Figure 8), as mentioned for the mycelium, and the WT and cultivated sporophores.

Table 4 summarizes the array of metabolites present in different stages of *H. erinaceus*.

The content of erinacine A in H.e.2 mycelium and of hericenone C and D in sporophores were measured by the calibration curves [10] (Table 5).

**Figure 9:**
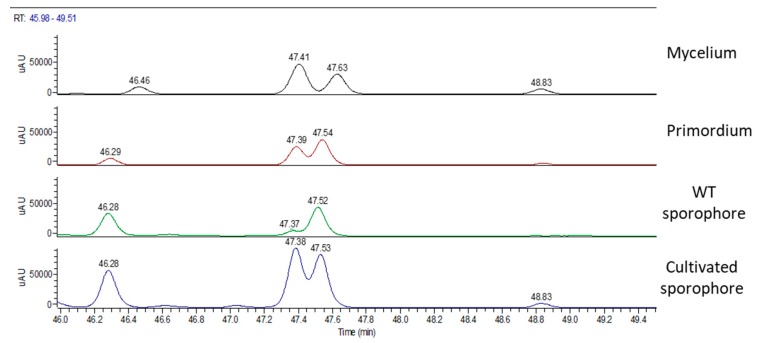
UV chromatographic traces of hericenes in (from top to bottom) mycelium, primordium, and wild type and cultivated sporophores.

Figure 9 summarizes the UV chromatographic traces of different samples where it is possible to identify peaks that are attributed to hericenes. From lower to higher RT, hericene D (RT at 46.47 or 42.29 or 46.28), hericene A (RT at 47.41 or 47.39 or 47.37 or 47.38), hericene B (RT at 47.63 or 47.54 or 47.52 or 47.53), and hericene C (RT at 48.83 or 48.84) were identified. As previously reported, hericenes were present in all the samples in different amounts.

Table 6 reports peak area values of hericenes A, B, C, and D in different samples.

## 3. Discussion

At present, medicinal mushrooms such as *H. erinaceus* are exploited as dietary foods or supplements, producing beneficial effects by daily use in a balanced and varied diet. There are different products available on the market, increasing in number year by year. Despite this, there are still unresolved issues, including standardization and safety for the production of fungal supplement. Standardization is still in its early stage because of the lack of protocols and international guidelines [32].

This study is placed within an interdisciplinary research project to draw up the steps for the production of high quality dietary supplements to improve cognitive functions. The project has been following all the stages of the supply chain: strains selection, production, extraction, chemical analysis, and finally a pre-clinical test on animal models. More specifically, this study is the first step planned to analyze the array of some metabolites present in different growth stages of the *H. erinaceus* collected in Italy.

Thanks to the comparison with standard molecules, we were able to identify and quantify erinacine A in mycelium, hericenones C and D, and in wild type (WT) and cultivated sporophores. 

In MicUNIPV, the Fungal Research Culture Collection at the University of Pavia (Italy), two strains of *H. erinaceus* collected in Italy are present to date: H.e.1 and H.e.2 [10,28]. The content of erinacine A in H.e.2 (105 µg/g) is slightly less compared to H.e.1 mycelium (150 µg/g) [10]. These amounts of erinacine A are comparable to that reported by Krzyczkowski et al. (2010) in improved submerged cultivation [33].

The same comparison between the WT sporophores showed that H.e.2 contains more hericenones C and D (760 µg/g and 100 µg/g, respectively) compared to H.e.1 (500 µg/g and <20 µg/g, respectively) [10]. These values are comparable with those of some strains reported by Lee et al. (2016) [18].

Therefore, given these data, the H.e.2 strain must be used for sporophore cultivation, whereas the H.e.1 for mycelium production. 

By comparing the WT and cultivated sporophores of H.e.2, hericenones C and D in the cultivated sporophores are about two folds higher (1560 µg/g vs. 760 µg/g and 188 µg/g vs. 100 µg/g, respectively). Generally, wild sporophores exhibit biological variability, depending on the different growth environment and on seasonality. Conversely, the cultivated conditions are more stable. In particular, for the mycelial colonization, for the appearance of primordia, and the development of sporophores, the medium components (nitrogen, carbon, and mineral sources) and environmental factors (pH, temperature, and relative humidity) are fundamental in order to optimize the growth and to influence the bioactive metabolites production.

The primordium is an intermediate stage of the fungus between the mycelium and the sporophore development, characterized by the formation of spider-like aerial spines that grow above the culture medium. Generally, there are still few studies concerning primordium and none for *H. erinaceus* [34,35]. In our primordium, only hericenes were present, without any hericenone and erinacine. Hericenes are also found in mycelium and sporophores, both WT and cultivated, in agreement to what was reported by Arnone et al. (1994) [30] and Kobayashi et al. (2018) [31]. Preliminarily, in order to obtain a relative measure among the different samples, we compared the peak areas of the single hericenes detected, which were supposed to be A, B, C, and D, by HPLC-UV-ESI/MS. In particular, it is notable that in H.e.2 mycelium and primordium, hericenes A and B are more present compared to hericenes C and D. In cultivated sporophores, all hericenes are present, with a wider peak area if compared to the other samples. In WT sporophores, hericene C is absent and hericene A is at a lower level, whereas hericenes B and D peak areas have considerable values. Hericene B is present in fairly constant quantities in all samples, whereas hericene C is present in smaller quantities.

It should be noted that the cultivated sporophore has higher content than all the hericenes compared to the WT one. Similarly, the contents of hericenones C and D are higher in the cultivated sporophores than in the WT.

We could hypothesize a chemical correlation between hericenes and hericenones. Hericene A has a side chain with palmitoyl acid similar to hericenone C. Hericene C is similar to hericenone D with a side chain with stearic acid. Hericene D is similar to hericenone H and contains a side chain with a linoleoyl acid. Thus, in these paired molecules the side chain is maintained but hericenones differ from hericenes for their oxidation state. We can speculate that hericenone C derives from the oxidation of hericene A and hericenone D from the oxidation of hericene C. Other hericenones, such as I and E, could derive from the oxidation of hericenes B and D, respectively.

Up to now, ethanolic extracts obtained from H.e.1 mycelium and sporophores, with the standardized amounts of erinacine A and hericenones C and D, have been used to evaluate the effects of oral supplementation on cognitive decline in a mice model, during physiological aging [10]. Because of the different amounts of the neuroactive metabolites present in the different strains, now it is possible to prepare the best extract blend for in vivo tests. The present study contributes as it re-addresses the selection of raw material. 

Further investigation will be carried out by setting different cultivation conditions to maximize the yield of bioactive metabolites.

## 4. Materials and Methods 

### 4.1. Study Area and Sampling

Samplings were conducted in the hilly area around Siena (Tuscany, Italy), where both Mediterranean and temperate environments are present. The plant communities are dominated by holm oak (*Quercus ilex*), strawberry tree (*Arbutus unedo*), heather (*Erica arborea*), Mediterranean buckthorn (*Rhamnus alaternus*), juniper (*Juniperus communis*), and other deciduous species such as the downy oak (*Q. pubescens*). *Q. ilex* is an important feature in the landscape, being usually prevalent and resistant to anthropic stress.

The wild type (WT) sporophore was collected from an old living specimen of *Q. ilex* and kept at 4 °C until experimental use.

### 4.2. H. erinaceus Samples for Chemical Analyses

The *H. erinaceus* samples processed for chemical analyses were: the WT sporophore (the sample was dried and maintained in a freezer at −20 °C for at least one month in order to avoid any further degradation); the strain isolated from it; the sporophores cultivated at the Botanical Garden of the University of Pavia (Italy) using the above mentioned isolated strain; the primordium that was the first aerial part consisting of mycelial cords. 

### 4.3. H. erinaceus Strain Isolation 

The isolation of mycelium in a pure culture from the WT was performed in accordance with the usual procedures [36,37,38]. Small pieces (up to 10 mm^3^) were aseptically cut off from the center of the WT and inoculated into Petri dishes containing 2% malt extract agar (MEA, Biokar Diagnostics). Chloramphenicol at 50 ppm was added in this first step. Incubation was performed at 24 °C in complete darkness. The isolated strain is maintained in the Fungal Research Culture Collection of Pavia University (MicUNIPV).

### 4.4. H. erinaceus Sporophores Cultivation

The cultivation of *H. erinaceus* sporophores was performed in the mushroom greenhouse of the Botanical Garden at the University of Pavia (Italy). As the substrate, a mix of 70% oak sawdust, 20% rice bran, and 10% wheat straw, combined with 1% sucrose and 1% calcium carbonate, was used [36,39,40,41]. The substrate was mixed and hydrated, and then 300 g were placed in polypropylene bags with filters to allow gas exchange. Each bag was sterilized twice at 120 °C for 60 min. 

In parallel, the spawn with *H. erinaceus* was prepared: the mycelium grew in sterilized polypropylene bags containing 300 g of hydrated barley. They were taken at 24 °C with 90% relative humidity (RH) in the dark for two weeks, until complete colonization.

We aseptically put and mixed 5% of spawn into each substrate bag. The cultivation room was kept at 24 °C and bathed to maintain high relative humidity (95%-100%).

Soon after the substrate was completely colonized by the mycelium, the bags were moved to a room where it was possible to carry out the light-dark cycle, maintaining the temperature at 18 °C–24 °C, the RH of 90%–95%, and good aeration condition to induce primordia formation.

In correspondence to the appearance of primordia, holes were made in the bags to allow the development of sporophores [36,39,40,41]. Once collected, the sporophores were weighed, measured, dried, and maintained frozen.

### 4.5. Extraction Procedures

The procedure of alcoholic extraction described by Lee et al. (2016) and Gerbec et al. (2015) [18,42] was followed with slight modification: 1 g of lyophilized mycelium/dried WT/cultivated sporophores/fresh primordium was blended with 10 mL of ethanol 70% and left in the thermostat at 50 °C for 24 h. At the end, the material was transferred for centrifugation (4000 rpm for 3 min) and the supernatant was stored at −20 °C for HPLC analysis. 

### 4.6. HPLC-UV-ESI/MS Method

HPLC-UV-ESI/MS analyses were carried out on a LCQ FLEET system (Thermo Fisher Scientific, San Jose, CA, USA), equipped with a PAD-UV detector working at 254 nm. The chromatographic separation was performed using an F5 HPLC column 150 × 3.0 mm, 2.7 μm particle size (Ascentis® Express, Merck KGaA, Darmstadt, Germany) maintained at 40 °C, with a flow rate of 0.3 mL/min and an injection volume of 20 µL. The mobile phase consisted of water containing 0.1% formic acid (solvent A) and acetonitrile (solvent B) (Table 7). The following gradient method was utilized: 0–9 min (30%–50% B), 9–27 min (50%–60% B), 27–54 min (60%–100% B), 54–69 min (100%–30% B), and 69–75 min (30% B). 

An Electro Spray Ionization (ESI) interface was used as an ion source, under positive ion conditions (ESI+). The Ion Spray voltage and Capillary voltage were set at 5 kV and 10 V in positive ion mode. The capillary temperature was 400 °C. Acquisition was performed both in Full Scan mode (mass range 200–2000 Da) and Dependent Scan mode. The data station utilized the Xcalibur MS Software Version 2.1.

Stock solutions of erinacine A and hericenones C and D (1 mg/mL) were prepared in 70% ethanol. Standard solutions with the final concentration range of 1–25 µg/mL for erinacine A and 20–100 µg/mL for hericenones C and D were obtained by the proper dilution of stock solutions.

Calibration curves were constructed by injecting the standard mixture solutions at five concentrations (1, 5, 10, 15, 25 µg/mL) for erinacine A and at four concentrations (20, 50, 75, 100 μg/mL) for hericenone C and D. Linear least-square regression analysis for the calibration curves showed correlation coefficients of 0.9968, 09945, and 0.9951, respectively, for erinacine A, hericenones C, and hericenones D with respect to the peak area, demonstrating a good linear relationship in the different ranges tested. Each concentration was analyzed in triplicate [10].

## 5. Conclusions

In this study, an array of metabolites at different growth stages of the fungus *H. erinaceus* collected in Italy was analyzed. In particular, for the first time we described the array of metabolites present in primordium stage, i.e., the hericenes. These molecules are also present from the formation of the mycelium to the appearance of the primordium and up to the sporophore development. Experiments in the future will focus on testing the functional role of these molecules in vitro and in vivo.

In conclusion, in our opinion this methodological approach is a necessary step for developing dietary supplements with a higher and standardized content of bioactive metabolites.

## Figures and Tables

**Table 1:** Characteristics of *Hericium erinaceus* wild type sporophore.

	*H. erinaceus*
fresh weight (g)	620
dried weight (g)	153
diameter (cm)	about 20
remarks on the sporophore	the collected specimen was mature, without any alteration by atmospheric or animal agents

**Table 2:** Molecular formula, chemical structures, and molecular weights of erinacine A and hericenones C and D.

	Erinacine A	Hericenone C	Hericenone D
molecular formula	C_25_H_36_O_6_	C_35_H_54_O_6_	C_37_H_58_O_6_
molecular weight (MW) (g/mol)	432	570	598
chemical structure	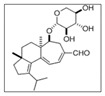	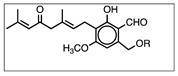 R = palmitoyl	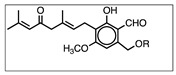 R = stearoyl

**Table 3:** Molecular formula, chemical structures, and molecular weights of hericenes.

	Hericene A	Hericene B	Hericene C	Hericene D
molecular formula	C_35_H_56_O_5_	C_37_H_58_O_5_	C_37_H_60_O_5_	C_37_H_56_O_5_
molecular weight (MW) (g/mol)	556	582	584	580
chemical structure	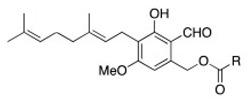			
	R = palmitoyl	R = oleoyl	R = stearoyl	R = lineoyl

**Table 4:** Presence of different molecules (erinacine A, hericenones C and D, and hericenes) in the lyophilized mycelium, fresh primordium, and dried sporophores.

	erinacine A	hericenone C	hericenone D	hericenes
mycelium	✓	-	-	✓
primordium	-	-	-	✓
WT sporophore	-	✓	✓	✓
cultivated sporophore	-	✓	✓	✓

**Table 5:** Content of erinacine A and hericenones C and D in H.e.2 lyophilized mycelium, fresh primordium and dried sporophores.

	Erinacine A (µg/g)	Hericenone C (µg/g)	Hericenone D (µg/g)
mycelium	105	-	-
primordium	-	-	-
WT sporophore	-	760	100
cultivated sporophore	-	1560	188

**Table 6:** Content of hericenes in mycelium, primordium, and wild type (WT) and cultivated sporophores.

	Total Area 10^3^	Hericene A Area 10^3^	Hericene B Area 10^3^	Hericene C Area 10^3^	Hericene D Area 10^3^
mycelium	684	327	232	51	74
primordium	557	201	262	21	73
WT sporophore	627	70	305	/	252
cultivated sporophore	1685	645	588	42	410

**Table 7:** The mobile phase and the gradient method.

Time	Solvent A	Solvent B
0	70	30
9	50	50
27	40	60
54	00	100
69	70	30
75	70	30
